# Supplementary material for: Loss of FLCN-FNIP1/2 induces a non-canonical interferon response in human renal tubular epithelial cells
Source: eLife. 2021 Jan 18;10:e61630. doi: 10.7554/eLife.61630 (PMC7899648; doi:10.7554/eLife.61630)
Supplement: Transparent reporting form [file elife-61630-transrepform.docx]

***eLife’s* transparent reporting form**

We encourage authors to provide detailed information *within their submission* to facilitate the interpretation and replication of experiments. Authors can upload supporting documentation to indicate the use of appropriate reporting guidelines for health-related research (see [EQUATOR Network](http://www.equator-network.org/%20)), life science research (see the [BioSharing Information Resource](https://biosharing.org/)), or the [ARRIVE guidelines](http://www.plosbiology.org/article/info:doi/10.1371/journal.pbio.1000412) for reporting work involving animal research. Where applicable, authors should refer to any relevant reporting standards documents in this form.

If you have any questions, please consult our Journal Policies and/or contact us: [editorial@elifesciences.org](mailto:editorial@elifesciences.org).

**Sample-size estimation**

- You should state whether an appropriate sample size was computed when the study was being designed
- You should state the statistical method of sample size computation and any required assumptions
- If no explicit power analysis was used, you should describe how you decided what sample (replicate) size (number) to use

Please outline where this information can be found within the submission (e.g., sections or figure legends), or explain why this information doesn’t apply to your submission:

No statistical methods were used to predetermine sample size. However, for RNAseq analyses we determined the value of using triplicate independent samples per biological condition (described in [Van Iterson *et al*., 2013](https://www.degruyter.com/view/journals/sagmb/12/4/article-p449.xml)). The used proteomic work flow, with two technical replicates per cell line, is extensively bench-marked for reproducibility (see Material and Methods section; [Piersma *et al.,* 2013](https://www.ncbi.nlm.nih.gov/pmc/articles/PMC3656797/)).

**Replicates**

- You should report how often each experiment was performed
- You should include a definition of biological versus technical replication
- The data obtained should be provided and sufficient information should be provided to indicate the number of independent biological and/or technical replicates
- If you encountered any outliers, you should describe how these were handled
- Criteria for exclusion/inclusion of data should be clearly stated
- High-throughput sequence data should be uploaded before submission, with a private link for reviewers provided (these are available from both GEO and ArrayExpress)

Please outline where this information can be found within the submission (e.g., sections or figure legends), or explain why this information doesn’t apply to your submission:

Independent FLCN^POS^ and FLCN^NEG^ cell lines were treated as biological replicates (although derived from the same cell line, each cell line is genetically different due to CRISPR mediated gene disruptions) and therefore allocated together in groups to perform FLCN dependent differential expression analyses.

The amount of performed experiments is described in the Materials and Methods section. The exact n per experiment is mentioned in each relevant figure legend.

All qPCR experiments were performed at least in duplicate (independent mRNA isolations) with three technical replicates per experiment. To determine quantitative gene expression data, levels were normalized to the geometric mean of two housekeeping genes.

Western blots were performed three or two times using lysates obtained from independent experiments.

Colony formation experiments were seeded in three technical replicates and repeated at least twice.

Amino acid starvation experiments, followed by immunofluorescent stainings and western blots were performed three times.

Supernatants for ELISA and CBA were measured twice in duplicate.

**Statistical reporting**

- Statistical analysis methods should be described and justified
- Raw data should be presented in figures whenever informative to do so (typically when N per group is less than 10)
- For each experiment, you should identify the statistical tests used, exact values of N, definitions of center, methods of multiple test correction, and dispersion and precision measures (e.g., mean, median, SD, SEM, confidence intervals; and, for the major substantive results, a measure of effect size (e.g., Pearson's r, Cohen's d)
- Report exact p-values wherever possible alongside the summary statistics and 95% confidence intervals. These should be reported for all key questions and not only when the p-value is less than 0.05.

Please outline where this information can be found within the submission (e.g., sections or figure legends), or explain why this information doesn’t apply to your submission:

Details of statistical analyses (tests, numbers of n, methods of multiple test correction, measure of effect size) are described in two distinct Materials & Methods sections:

1) *Differential expression analysis of RNAseq data*

The R package edgeR (Robinson, McCarthy, & Smyth, 2010) was used to compare RNA-seq profiles between FLCN^POS^ and FLCN^NEG^ replicates, as well as between TP53^POS^ and TP53^NEG^ replicates. This involved reading in the gene-level counts, computing library-size normalizing factors using the trimmed-mean of M-values (TMM) method and then fitting a model to estimate the group effect. Obtained p-values were corrected for multiple testing using the Benjamini-Hochberg false discovery rate (FDR) step-up procedure (Benjamini & Hochberg, 1995). Significant P-values are indicated in figures as *≤0.05, **≤0.01, ***≤0.001, ****≤0.0001.

*2) Protein identification & Label‐free quantitation*

Raw counts were normalized on the sum of spectral counts for all identified proteins in a particular sample, relative to the average sample sum determined with all samples. To find statistically significant differences in normalized counts between sample groups, we applied the beta‐binomial test (Pham, Piersma, Warmoes, & Jimenez, 2010), which takes into account within‐sample and between‐sample variation using an alpha level of 0.05.

Complete transcriptomic and proteomic data sets, including exact p-values, are provided (see section Source data for deposit details).

Exact n per experiment is mentioned in each relevant figure legend and manuscript text. Also, when n per group is less than 10, we present individual values as dots in bar graphs (Figure 6 and S6). Significant P-values are indicated as *≤0.05, **≤0.01, ***≤0.001, ****≤0.0001.

(For large datasets, or papers with a very large number of statistical tests, you may upload a single table file with tests, Ns, etc., with reference to sections in the manuscript.)

**Group allocation**

- Indicate how samples were allocated into experimental groups (in the case of clinical studies, please specify allocation to treatment method); if randomization was used, please also state if restricted randomization was applied
- Indicate if masking was used during group allocation, data collection and/or data analysis

Please outline where this information can be found within the submission (e.g., sections or figure legends), or explain why this information doesn’t apply to your submission:

Samples were allocated into groups based on their knock-out status. No randomization was required. Samples were treated according to the same protocols side-by-side with the respective controls. Methods used for group to group comparisons are explained in the paper and materials and methods section.

**Additional data files (“source data”)**

- We encourage you to upload relevant additional data files, such as numerical data that are represented as a graph in a figure, or as a summary table
- Where provided, these should be in the most useful format, and they can be uploaded as “Source data” files linked to a main figure or table
- Include model definition files including the full list of parameters used
- Include code used for data analysis (e.g., R, MatLab)
- Avoid stating that data files are “available upon request”

Please indicate the figures or tables for which source data files have been provided:

Transcriptomic and proteomic data shown in Figure 2B and 2C is attached as Supplementary_Table1_Glykofridis et al.

Raw RNAseq count data from RPTEC controls and FLCN knock out cells (Figure 1 and Figure 2), generated in this study, has been deposited in Dryad under accession number doi:10.5061/dryad.6djh9w0zs.

Proteomic data has been deposited in ProteomeXchange database under accession number PXD021346.

Unique identifiers for the publicly available datasets used are indicated, and source data other than those provided in the Article or Supplementary Information are available from the corresponding author upon reasonable request.
